# Critique of a Radically New Model for Plasma Membrane Bilayer Organization

**DOI:** 10.1002/bies.70114

**Published:** 2026-02-11

**Authors:** Yvonne Lange, Theodore L. Steck

**Affiliations:** ^1^ Department of Pathology Rush University Medical Center Chicago Illinois USA; ^2^ Department of Biochemistry and Molecular Biology University of Chicago Chicago Illinois USA

**Keywords:** asymmetry, bilayer, cholesterol, erythrocyte, membrane, model, phospholipid, sidedness

## Abstract

A novel model recently proposed by Doktorova et al. challenges current concepts for the molecular organization of the plasma membrane bilayer. It is at odds with previously published research. The model posits that there are far fewer phospholipid molecules in the outer leaflet of the bilayer than in the inner leaflet and that the resulting area deficit is filled by cholesterol. This conclusion is based on the incomplete hydrolysis of the phosphatidylcholine in intact erythrocytes by phospholipase A2, leading to the inference that the undigested fraction is endofacial. But the incomplete digestion can be explained by product inhibition. Furthermore, ultrastructural analysis has shown that almost all of the phosphatidylcholine in the erythrocyte bilayer resides in the outer leaflet. Finally, the high concentration of cholesterol predicted for the outer leaflet of resting human erythrocytes is not detectable by probes. The conflict of the new model with the literature makes it insecure.

## Graphical abstract

We critique a model by Doktorova et al. (doi.org/10.1016/j.cell.2025.02.034) that challenges textbook depictions of the distribution of lipids in plasma membrane bilayers. They propose that the outer leaflet has about half as many phospholipids as the inner leaflet (left figure) and is compensated for by an excess of cholesterol (right figure).

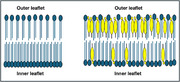

## 1 Introduction

It is generally held that eukaryotic plasma membranes are built upon a bilayer of phospholipids with glycolipids, sterols, and proteins integrated therein. The asymmetrical transbilayer disposition of individual proteins and the exofacial location of glycolipids are well established [1, 2, 3]. The transverse asymmetry of human erythrocyte plasma membrane phospholipids has also been characterized [4]. Nearly all of the sphingomyelin is accessible to probes in the intact cell; hence, it is exposed at the exofacial side. Phosphatidylethanolamine, phosphatidylserine, and phosphatidylinositol and its derivatives resist cleavage by phospholipase A2 and have therefore been assigned to the cytoplasmic leaflet [1, 5, 6, 7, 8, 9]. On the other hand, the transbilayer distributions of both phosphatidylcholine and cholesterol are unsettled [1, 10, 11].

**FIGURE 1:**
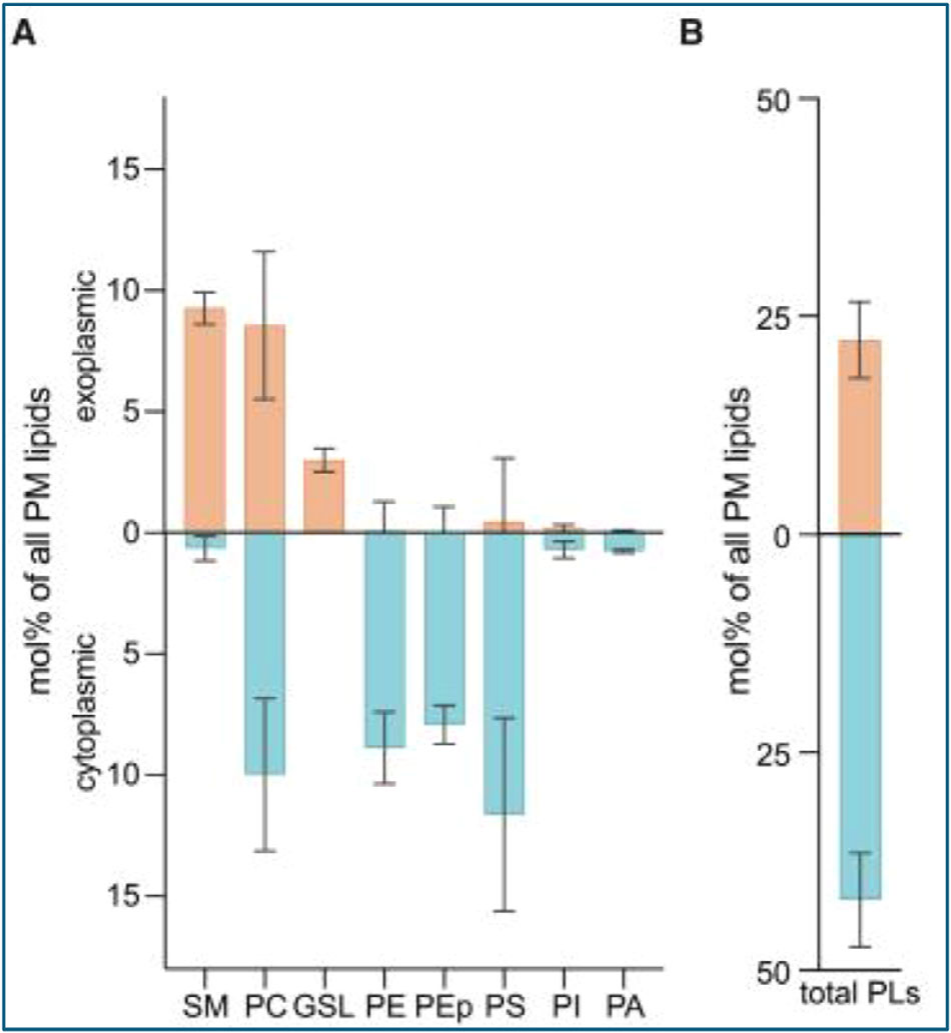
Proposed transbilayer distribution of the polar lipids in the human erythrocyte membrane [9]. Intact cells were digested with lipases. The fraction of each lipid that was hydrolyzed was assigned to the exofacial leaflet and the remainder to the contralateral leaflet. Cholesterol is not shown but is included in the total plasma membrane lipids. Note that more than half of the phosphatidylcholine is assigned to the cytoplasmic leaflet. Reproduced from Doktorova et al. with permission. GSL, glycosphingolipid; PA, phosphatidic acid; PC, phosphatidylcholine; PE, phosphatidylethanolamine; PEp, PE plasmalogen; PI, phosphatidylinositol; PL, phospholipid; PM, plasma membrane; PS, phosphatidylserine; SM, sphingomyelin.

A recent publication by Doktorova et al. presents a new model for plasma membrane bilayer organization that differs dramatically from current concepts [9]. It derives from their determination that only about half of the phosphatidylcholine in intact red cell membranes is hydrolyzed by phospholipase A2 (Figure 1). Four key inferences were drawn from this finding: (a) The undigested phosphatidylcholine resides in the cytoplasmic leaflet of the bilayer. (b) This result was taken to mean that the number of phospholipid molecules in the endofacial leaflet greatly exceeds that on the exofacial side, possibly by as much as two‐fold. (c) It follows that there is a large difference in the total cross‐sectional area of the phospholipids in the two leaflets. (d) Given that the two leaflets have essentially equal surface areas overall, it was proposed that the gap in the outer leaflet is filled by cholesterol [9]. Simulations predicted that ∼77% of the bilayer sterol is in the exofacial leaflet and ∼23% is in the contralateral leaflet.

## 2 Countervailing Literature

The model advanced by Doktorova et al. for the molecular organization of the plasma membrane bilayer [9] is at odds with the following three lines of evidence, none of which they addressed.

### 2.1 Essentially All of the Phosphatidylcholine is Exofacial

**FIGURE 2:**
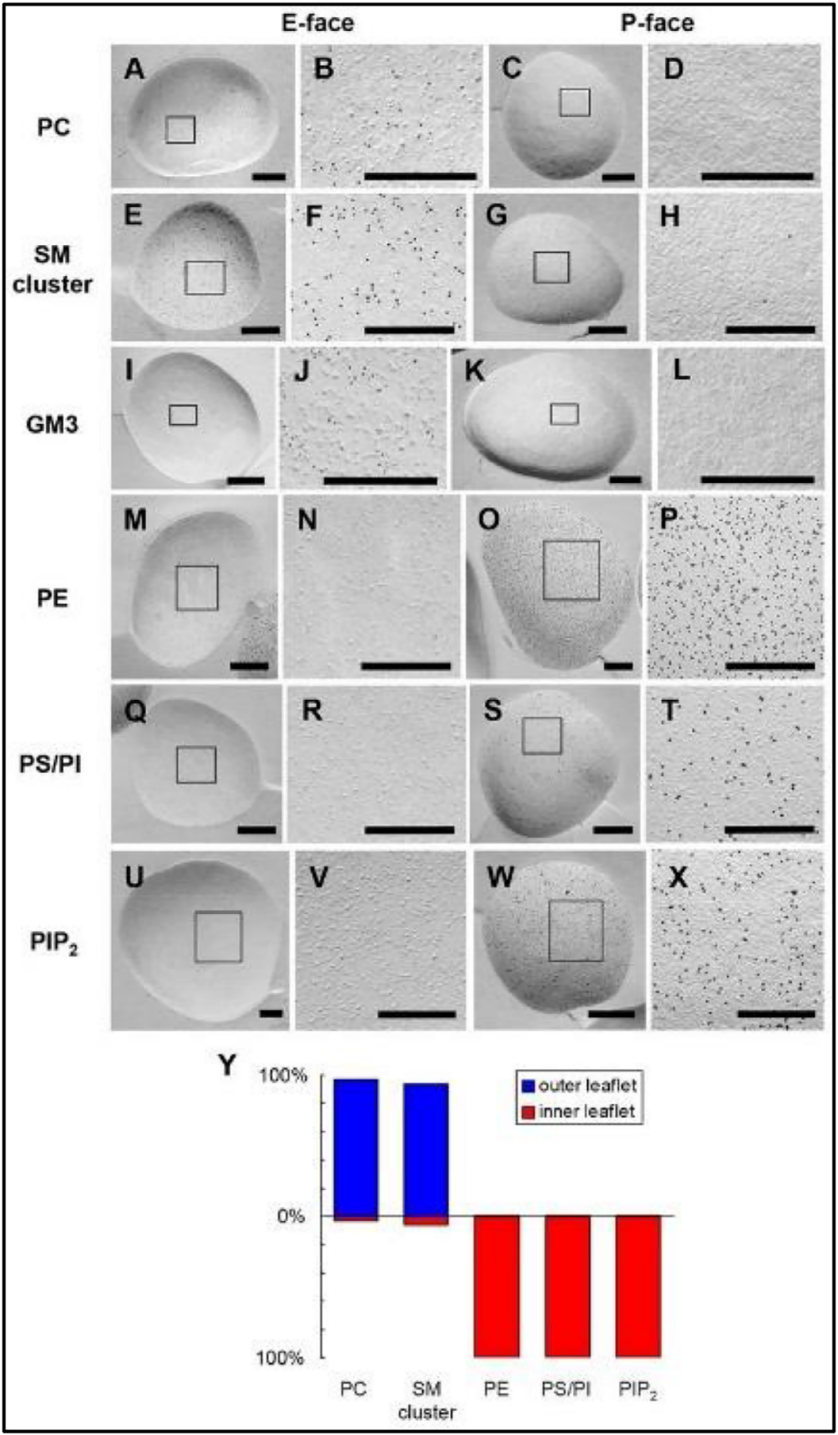
Ultrastructural analysis of the distribution of polar lipids between the two leaflets of the human erythrocyte bilayer. The membranes were cleaved by freeze‐fracture and decorated with the indicated lipid‐specific probes. The E‐face shows the outer leaflet, and the P‐face shows the inner leaflet at both low and high magnification. The relevant finding is that nearly all of the phosphatidylcholine was found in the outer leaflet. Reproduced from Murate et al. with permission [6]. GM3, ganglioside GM3; PC, phosphatidylcholine; PIP2, phosphatidylinositol 4,5‐bisphosphate; SM, sphingomyelin.

The distribution of polar lipids between the two leaflets of the red cell plasma membrane bilayer has been analyzed with specific immuno‐gold tagged protein probes using “sodium dodecyl sulfate‐digested freeze‐fracture replica labeling” or SDS‐F [6, 12]. Telling electron microscope images are reproduced in Figure 2. Concordant with many enzyme digestion studies, this technique placed all of the phosphatidylserine, phosphatidylethanolamine, phosphatidylinositol and phosphatidylinositol 4,5‐bisphosphate in the inner leaflet. In addition, almost all of the membrane sphingomyelin and ganglioside GM3 were found in the outer leaflet. Crucially, nearly all of the phosphatidylcholine was exofacial.

The diametrical distribution of each of the lipids between the two leaflets in these studies provides an internal control. Furthermore, the fidelity of the technique was corroborated with liposomes composed of different saturated and unsaturated phosphatidylcholines as well as other phospholipids [6, 12, 13]. In particular, control experiments showed that anionic phospholipids do not interfere with the labeling of phosphatidylcholine in vesicles. This lends assurance that the detection of phosphatidylcholine at the endofacial surface of the red cell membrane would not have been hindered by the negatively charged phospholipids therein [6]. These ultrastructural data contrast strongly with the phospholipase digestion results which were taken to signify that half or more of the phosphatidylcholine is located in the cytoplasmic leaflet of the membrane.

### 2.2 Why Does Plasma Membrane Phosphatidylcholine Resist Digestion?

Doktorova et al. determined that about half of the phosphatidylcholine in intact red cells was not hydrolyzed by phospholipase A2 and proposed that it must therefore be endofacial (Figure 1) [8, 9]. However, there is a sampling bias in this approach: probing just the outer leaflet of the plasma membrane bilayer risks underestimating any unreacted exofacial phospholipid. Indeed, in an early study, a combination of sphingomyelinase and phospholipase A2 hydrolyzed 48% of the phospholipid in intact red cells, positive evidence that there is no phospholipid deficit in the outer leaflet.

**FIGURE 3:**
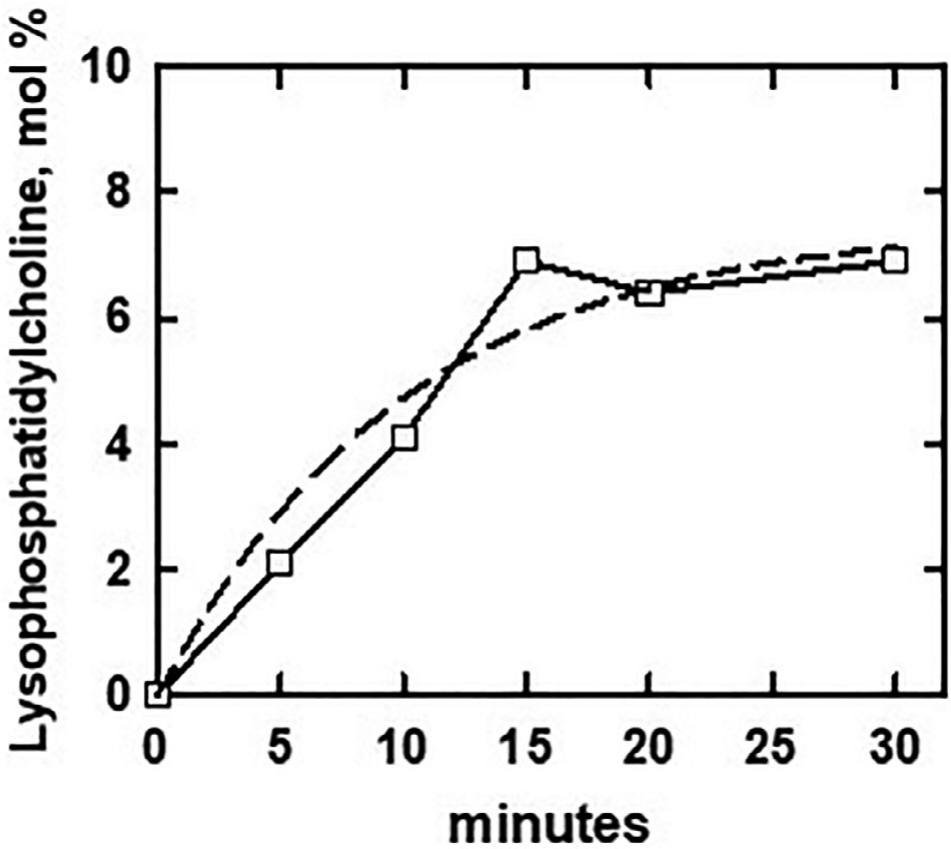
Hydrolysis of the phosphatidylcholine in intact human erythrocytes by phospholipase A2. The data for lysophosphatidylcholine production were replotted from Figure S1E in Lorent et al. [8]. For comparison, we generated the dashed curve assuming Michaelis–Menton kinetics. The question at issue is whether the flattening of the experimental curve at 15 min is due to product (or another type of) inhibition rather than exhaustion of the exofacial substrate as proposed [8, 9].

The large variability of the undigested fraction suggests an experimental rather than a biological cause for the observed incomplete hydrolysis of phosphatidylcholine (Figure 1) [9]. Furthermore, factors other than the sidedness of the phosphatidylcholine can limit its digestion [14, 15, 16, 17, 18, 19, 20]. In particular, product inhibition can prematurely curtail the attack of phospholipase A2 on its substrate [21]. This effect has been observed for the honeybee enzyme used by Doktorova et al. [20, 22, 23]. Indeed, the progress curve for digestion underlying the study in question suggests product inhibition [8]. That is, the time course appears to show a more abrupt deceleration than is predicted for Michaelis–Menton kinetics (Figure 3). (The reaction also shows a slight initial acceleration ascribable to early product activation of the enzyme [17, 24].) Both chemical and physical mechanisms have been proposed to explain product inhibition. In the former case, the products of phospholipid hydrolysis are competitive inhibitors of the enzyme [21, 25, 26, 27, 28]. The latter mechanism implicates the increase in membrane surface pressure imposed by the hydrolysis products: compressing the bilayer makes the substrate less vulnerable to further attack [20, 29, 30].

Our analysis of the primary data in Figure 3 does not settle the question of product inhibition. A more definitive time course is needed. In addition, future work could test for the putative undigested phosphatidylcholine in the outer leaflet of the intact cell. Adding sphingomyelinase to the phospholipase A2 increases the digestion of phosphatidylcholine in intact red cells. In one such study, the phosphatidylcholine was hydrolyzed almost as completely as was the sphingomyelin: 76% versus 82% [31]. Depletion of plasma membrane cholesterol with methyl‐β‐cyclodextrin might also increase the susceptibility of the phosphatidylcholine [19]. In addition, serum albumin can relieve product inhibition by sequestering the inhibitory digestion products [21]. (However, their removal might promote hemolysis.)

Another factor limiting the action of phospholipase A2 on the exofacial face of plasma membrane bilayers is the close packing of their saturated phospholipids [8, 14, 32, 33]. If, as we suspect, the exofacial leaflet is replete in saturated phosphatidylcholines that resist digestion, the extent of chain saturation and tight packing in the outer leaflet would be even greater than proposed [8].

In any case, why phospholipase A2 digestion routinely fails to hydrolyze much of the plasma membrane phosphatidylcholine is presently uncertain. This makes the conclusion of Doktorova et al. premature [9].

### 2.3 Evidence for Excess Cholesterol at the Erythrocyte Surface is Lacking

The cholesterol in monolayers, vesicles, and plasma membranes is almost entirely complexed with the phospholipids [34, 35, 36, 37, 38]. These studies showed that only cholesterol exceeding stoichiometric equivalence is readily accessible to ligands and probes such as cholesterol oxidase, methyl‐β‐cyclodextrin, and bacterial toxins. It can be estimated from the data presented by Doktorova et al. [9] that the cholesterol/phospholipid ratio in the outer leaflet of the red cell bilayer is ∼1.45 mole/mole (see Box 1). This high level of cholesterol should be readily detected at the surface of unperturbed human erythrocytes. However, it is not [11, 36, 39, 40, 41].

Doktorova et al. did a nice experiment to estimate the transverse distribution of sterols [9]. They labeled erythrocytes with a small amount of dehydroergosterol (DHE), a good cholesterol surrogate and reporter that presumably equilibrates across the bilayer. The addition of Di4, a cationic fluorescence quencher that cannot cross the bilayer, reduced the DHE fluorescence in the bilayer by about two‐thirds. This is taken as the fraction of DHE (and, presumably, cholesterol) located in the outer leaflet, supporting their premise that about three‐quarters of the sterol is exofacial.

**BOX 1 | Calculation of leaflet cholesterol/phospholipid ratios from Doktorova et al. [9]**

1. The average mole ratio of inner leaflet (i) to outer leaflet (o) phospholipid (PL) is ∼1.8 (their Figure 1D). The inner leaflet therefore has 64.3%, and the outer leaflet has 35.7% of the total phospholipid (PL_t_). For 100 moles of total phospholipid, PL_i_ = 64.3 and PL_o_ = 35.7 moles.
2. Cholesterol (CH) constitutes 40 mol% of the total membrane lipid [i.e.,100 × CH_t_/(CH_t_ + PL_t_)]. This corresponds to CH_t_/PL_t_ = 0.67. That is, the concentration of membrane cholesterol is 67 moles/100 moles phospholipid.
3. The molecular dynamics simulation predicts that 77% of the cholesterol is exofacial and 23% is endofacial. Thus, CH_o_ = 51.6 and CH_i_ = 15.4 moles per 100 moles phospholipid.
4. It follows that the lipid mole ratio in the outer leaflet is CH_o_/PL_o_ = 51.6/35.7 = 1.45 and the lipid mole ratio in the inner leaflet is CH_i_/PL_i_ = 15.4/64.3 = 0.24.

Other mechanisms might lead to excess exofacial cholesterol [8, 10, 11, 42, 43, 44, 45]. In some studies, the driver of the transbilayer asymmetry of the sterol is taken to be its preferential association with the saturated phospholipids abundant in the outer leaflet rather than the surface area compensation postulated by Doktorova et al. [9]. The association of the cholesterol with the saturated phospholipids in the exofacial leaflet should reduce its chemical activity and therefore its reactivity with probes. This is not predicted by the Doktorova model [9] but is found experimentally [9, 11, 36, 39, 40, 41].

## 3 Conclusions

Three lines of evidence counter the proposal by Doktorova et al. that a large fraction of the phosphatidylcholine in the erythrocyte plasma membrane resides in its endofacial leaflet (Figure 1) and that the imbalance in phospholipid area across the bilayer is compensated for by an excess of exofacial cholesterol. First, an ultrastructural analysis of the binding of phospholipid‐specific ligands showed that almost all of the phosphatidylcholine in this membrane is in its outer leaflet (Figure 2). Second, both the phospholipase A2 literature and the time course of digestion reported by Lorent et al. (Figure 3) suggest that the limited hydrolysis of phosphatidylcholine they observed might not signify its sequestration in the cytoplasmic leaflet but rather its incomplete digestion at the exofacial surface. Third, the high level of uncomplexed cholesterol they proposed for the outer leaflet of the resting human erythrocyte should be readily detectable by various probes, but it is not.

One more consideration: in sheep erythrocytes, the phosphatidylcholine is almost completely replaced by sphingomyelin [46, 47, 48, 49]. This lipid constitutes about half of the total phospholipid complement in these cells. If, as reviewed above, sphingomyelin is confined to the outer leaflet of the erythrocyte bilayer, sheep red cells would have no exofacial area deficit and, therefore, no sterol excess.

Doktorova et al. did not address these issues. Rather, they supported their model with a variety of ancillary experiments and conceptual tests [9]. Those data and insights are valuable. However, they do not rule out other models for the distribution of lipids across the plasma membrane bilayer and are not sufficient to establish theirs.

## Author Contributions

The authors contributed equally to the conception and writing of the manuscript.

## Funding

The authors have nothing to report.

## Conflicts of Interest

The authors declare no conflicts of interest.

## Data Availability

Data sharing is not applicable to this article as no new data were created or analyzed in this study.

## References

[1] M. Murate and T. Kobayashi , “Revisiting Transbilayer Distribution of Lipids in the Plasma Membrane,” Chemistry and Physics of Lipids 194 (2016): 58–71. 26319805 10.1016/j.chemphyslip.2015.08.009

[2] P. Mattjus , “Specificity of the Mammalian Glycolipid Transfer Proteins,” Chemistry and Physics of Lipids 194 (2016): 72–78. 26234207 10.1016/j.chemphyslip.2015.07.018

[3] G. Pabst and S. Keller , “Exploring Membrane Asymmetry and Its Effects on Membrane Proteins,” Trends in Biochemical Sciences 49, no. 4 (2024): 333–345. 38355393 10.1016/j.tibs.2024.01.007

[4] J. Op den Kamp , “Lipid Asymmetry in Membranes,” Annual Review of Biochemistry 48 (1979): 47–71. 10.1146/annurev.bi.48.070179.000403 382989

[5] A. Zachowski , “Phospholipids in Animal Eukaryotic Membranes—Transverse Asymmetry and Movement,” Biochemical Journal 294 (1993): 1–14. 8363559 10.1042/bj2940001 PMC1134557

[6] M. Murate , M. Abe , K. Kasahara , K. Iwabuchi , M. Umeda , and T. Kobayashi , “Transbilayer Distribution of Lipids at Nano Scale,” Journal of Cell Science 128, no. 8 (2015): 1627–1638. 25673880 10.1242/jcs.163105

[7] R. Clarke , K. Hossain , and K. Cao , “Physiological Roles of Transverse Lipid Asymmetry of Animal Membranes,” Biochimica et Biophysica Acta ‐ Biomembranes 1862, no. 10 (2020): 183382. 32511979 10.1016/j.bbamem.2020.183382

[8] J. H. Lorent , K. R. Levental , L. Ganesan , et al., “Plasma Membranes Are Asymmetric in Lipid Unsaturation, Packing and Protein Shape,” Nature Chemical Biology 16, no. 6 (2020): 644–652. 32367017 10.1038/s41589-020-0529-6 PMC7246138

[9] M. Doktorova , J. L. Symons , X. Zhang , et al., “Cell Membranes Sustain Phospholipid Imbalance via Cholesterol Asymmetry,” Cell 188, no. 10 (2025): 2586–2602. 40179882 10.1016/j.cell.2025.02.034 PMC12085300

[10] T. Steck and Y. Lange , “Transverse Distribution of Plasma Membrane Bilayer Cholesterol: Picking Sides,” Traffic (Copenhagen, Denmark) 19, no. 10 (2018): 750–760. 29896788 10.1111/tra.12586

[11] T. Steck and Y. Lange , “Determination of the Transbilayer Distribution of Plasma Membrane Cholesterol,” preprint, bioRxiv, November 14, 2025: 2025.11.13.687888.

[12] K. Fujimoto , M. Umeda , and T. Fujimoto , “Transmembrane Phospholipid Distribution Revealed by Freeze‐fracture Replica Labeling,” Journal of Cell Science 109, no. 10 (1996): 2453–2460. 8923206 10.1242/jcs.109.10.2453

[13] K. S. Nam , K. Igarashi , M. Umeda , and K. Inoue , “Production and Characterization of Monoclonal Antibodies That Specifically Bind to Phosphatidylcholine,” Biochimica et Biophysica Acta (BBA)—Lipids and Lipid Metabolism 1046, no. 1 (1990): 89–96. 1697768 10.1016/0005-2760(90)90098-i

[14] D. Shah and J. Schulman , “Enzymic Hydrolysis of Various Lecithin Monolayers Employing Surface Pressure and Potential Technique,” Journal of Colloid and Interface Science 25, no. 1 (1967): 107–119.

[15] B. Roelofsen , R. Zwaal , P. Comfurius , C. Woodward , and L. Van Deenen , “Action of Pure Phospholipase A 2 and Phospholipase C on human Erythrocytes and Ghosts,” Biochimica Et Biophysica Acta 241, no. 3 (1971): 925–929. 5003696 10.1016/0005-2736(71)90024-1

[16] M. Adamich and E. Dennis , “Exploring the Action and Specificity of Cobra Venom Phospholipase A2 Toward Human Erythrocytes, Ghost Membranes, and Lipid Mixtures,” Journal of Biological Chemistry 253, no. 14 (1978): 5121–5125. 670181

[17] M. Jain and O. Berg , “The Kinetics of Interfacial Catalysis by Phospholipase A2 and Regulation of Interfacial Activation: Hopping Versus Scooting,” Biochimica et Biophysica Acta (BBA)—Lipids and Lipid Metabolism 1002, no. 2 (1989): 127–156. 2649150 10.1016/0005-2760(89)90281-6

[18] L. B. Jensen , N. K. Burgess , D. D. Gonda , et al., “Mechanisms Governing the Level of Susceptibility of Erythrocyte Membranes to Secretory Phospholipase A2,” Biophysical Journal 88, no. 4 (2005): 2692–2705. 15681653 10.1529/biophysj.104.056457 PMC1305365

[19] A. L. Heiner , E. Gibbons , J. L. Fairbourn , et al., “Effects of Cholesterol on Physical Properties of human Erythrocyte Membranes: Impact on Susceptibility to Hydrolysis by Secretory Phospholipase A2,” Biophysical Journal 94, no. 8 (2008): 3084–3093. 18192373 10.1529/biophysj.107.118356 PMC2275687

[20] G. Feigenson , “Spiers Memorial Lecture: Experimental Discovery of Asymmetric Bilayers, and a Recent Asymmetry Example,” Faraday Discussions 259, no. 0 (2025): 9–25. 40314219 10.1039/d5fd00041f

[21] J. Kupferberg , S. Yokoyama , and F. Kézdy , “The Kinetics of the Phospholipase A2‐catalyzed Hydrolysis of Egg Phosphatidylcholine in Unilamellar Vesicles. Product Inhibition and Its Relief by Serum Albumin,” Journal of Biological Chemistry 256, no. 12 (1981): 6274–6281. 7240204

[22] R. Yunes , A. Goldhammer , W. K. Garner , and E. Cordes , “Phospholipases—MELITTIN Facilitation of Bee Venom Phospholipase A2‐Catalyzed Hydrolysis of Unsonicated Lecithin Liposomes,” Archives of Biochemistry and Biophysics 183, no. 1 (1977): 105–112. 20842 10.1016/0003-9861(77)90424-6

[23] K. Conricode and R. Ochs , “Mechanism for the Inhibitory and Stimulatory Actions of Proteins on the Activity of Phospholipase‐A2,” Biochimica Et Biophysica Acta 1003, no. 1 (1989): 36–43. 2469472 10.1016/0005-2760(89)90095-7

[24] A. Raudino , “A Model for the Enzyme Activity in Systems with Large Composition Fluctuations. An Application to the Unusual Kinetics of Phospholipase A2,” European Physical Journal B—Condensed Matter and Complex Systems 2, no. 2 (1998): 197–210.

[25] A. Plückthun and E. Dennis , “Activation, Aggregation, and Product Inhibition of Cobra Venom Phospholipase A2 and Comparison With Other Phospholipases,” Journal of Biological Chemistry 260, no. 20 (1985): 11099–11106. 4030786

[26] T. Yasuda , J. Hirohara , T. Okumura , and K. Saito , “Purification and Characterization of Phospholipase‐A2 From Rat Stomach,” Biochimica Et Biophysica Acta 1046, no. 2 (1990): 189–194. 2223858 10.1016/0005-2760(90)90188-4

[27] B. Lathrop and R. Biltonen , “Calcium and Magnesium Dependence of Phospholipase‐A2‐Catalyzed Hydrolysis of Phosphatidylcholine Small Unilamellar Vesicles,” Journal of Biological Chemistry 267, no. 30 (1992): 21425–21431. 1400456

[28] T. J. Cunningham , L. Yao , and A. Lucena , “Product Inhibition of Secreted Phospholipase A2 May Explain Lysophosphatidylcholines' Unexpected Therapeutic Properties,” Journal of Inflammation 5, no. 1 (2008): 17. 18945345 10.1186/1476-9255-5-17 PMC2580763

[29] R. Demel , W. Geurts van Kessel , R. Zwaal , B. Roelofsen , and L. van Deenen , “Relation Between Various Phospholipase Actions on Human Red Cell Membranes and the Interfacial Phospholipid Pressure in Monolayers,” Biochimica Et Biophysica Acta 406, no. 1 (1975): 97–107. 1174576 10.1016/0005-2736(75)90045-0

[30] R. Zwaal , B. Roelofsen , P. Comfurius , and L. van Deenen , “Organization of Phospholipids in Human Red Cell Membranes as Detected by the Action of Various Purified Phospholipases,” Biochimica Et Biophysica Acta 406, no. 1 (1975): 83–96. 169915 10.1016/0005-2736(75)90044-9

[31] A. Verkleij , R. Zwaal , B. Roelofsen , P. Comfurius , D. Kastelijn , and L. van Deenen , “The Asymmetric Distribution of Phospholipids in the Human Red Cell Membrane. A Combined Study Using Phospholipases and Freeze‐Etch Electron Microscopy,” Biochimica et Biophysica Acta (BBA)—Biomembranes 323, no. 2 (1973): 178–193. 4356540 10.1016/0005-2736(73)90143-0

[32] R. Verger , J. Rietsch , M. C. Van Dam‐Mieras , and G. H. de Haas , “Comparative Studies of Lipase and Phospholipase A2 Acting on Substrate Monolayers,” Journal of Biological Chemistry 251, no. 10 (1976): 3128–3133. 1270439

[33] A. Gupta , T. Korte , A. Herrmann , and T. Wohland , “Plasma Membrane Asymmetry of Lipid Organization: Fluorescence Lifetime Microscopy and Correlation Spectroscopy Analysis,” Journal of Lipid Research 61, no. 2 (2020): 252–266. 31857388 10.1194/jlr.D119000364 PMC6997606

[34] H. McConnell and A. Radhakrishnan , “Condensed Complexes of Cholesterol and Phospholipids,” Biochimica Et Biophysica Acta 1610, no. 2 (2003): 159–173. 12648771 10.1016/s0005-2736(03)00015-4

[35] Y. Lange and T. Steck , “Cholesterol Homeostasis and the Escape Tendency (Activity) of Plasma Membrane Cholesterol,” Progress in Lipid Research 47, no. 5 (2008): 319–332. 18423408 10.1016/j.plipres.2008.03.001 PMC2659507

[36] Y. Lange , S. M. A. Tabei , J. Ye , and T. L. Steck , “Stability and Stoichiometry of Bilayer Phospholipid–Cholesterol Complexes: Relationship to Cellular Sterol Distribution and Homeostasis,” Biochemistry 52, no. 40 (2013): 6950–6959. 24000774 10.1021/bi400862q PMC3859718

[37] J. P. Litz , N. Thakkar , T. Portet , and S. L. Keller , “Depletion With Cyclodextrin Reveals Two Populations of Cholesterol in Model Lipid Membranes,” Biophysical Journal 110, no. 3 (2016): 635–645. 26840728 10.1016/j.bpj.2015.11.021 PMC4744159

[38] Y. Lange and T. Steck , “Active Cholesterol 20 Years On,” Traffic (Copenhagen, Denmark) 21, no. 11 (2020): 662–674. 32930466 10.1111/tra.12762

[39] Y. Lange , H. Cutler , and T. Steck , “The Effect of Cholesterol and Other Intercalated Amphipaths on the Contour and Stability of the Isolated Red Cell Membrane,” Journal of Biological Chemistry 255, no. 19 (1980): 9331–9337. 7410427

[40] R. S. Chakrabarti , S. A. Ingham , J. Kozlitina , et al., “Variability of Cholesterol Accessibility in Human Red Blood Cells Measured Using a Bacterial Cholesterol‐binding Toxin,” Elife 6 (2017): 23355. 10.7554/eLife.23355 PMC5323040 28169829

[41] A. Ayuyan and F. Cohen , “The Chemical Potential of Plasma Membrane Cholesterol: Implications for Cell Biology,” Biophysical Journal 114, no. 4 (2018): 904–918. 29490250 10.1016/j.bpj.2017.12.042 PMC5984996

[42] D. Allender , A. Sodt , and M. Schick , “Cholesterol‐Dependent Bending Energy Is Important in Cholesterol Distribution of the Plasma Membrane,” Biophysical Journal 116, no. 12 (2019): 2356–2366. 31023537 10.1016/j.bpj.2019.03.028 PMC6589153

[43] M. Aghaaminiha , A. M. Farnoud , and S. Sharma , “Quantitative Relationship Between Cholesterol Distribution and Ordering of Lipids in Asymmetric Lipid Bilayers,” Soft Matter 17, no. 10 (2021): 2742–2752. 33533367 10.1039/d0sm01709d

[44] M. Varma and M. Deserno , “Distribution of Cholesterol in Asymmetric Membranes Driven by Composition and Differential Stress,” Biophysical Journal 121, no. 20 (2022): 4001–4018. 35927954 10.1016/j.bpj.2022.07.032 PMC9674969

[45] T. Yamada and W. Shinoda , “Asymmetry and Heterogeneity in the Plasma Membrane,” Biophysical Journal 125, no. 2 (2026): 387–395.40556323 10.1016/j.bpj.2025.06.026

[46] M. Llanillo , J. Sánchez‐Yagüe , A. Checa , E. M. Martín‐Valmaseda , and A. Felipe , “Phospholipid and Fatty Acid Composition in Stored Sheep Erythrocytes of Different Densities,” Experimental hematology 23, no. 3 (1995): 258–264. 7875242

[47] G. Nelson , “Lipid Composition of Erythrocytes in Various Mammalian Species,” Biochimica et Biophysica Acta (BBA)—Lipids and Lipid Metabolism 144, no. 2 (1967): 221–232. 6064604 10.1016/0005-2760(67)90152-x

[48] J. Florin‐Christensen , C. E. Suarez , M. Florin‐Christensen , et al., “A Unique Phospholipid Organization in Bovine Erythrocyte Membranes,” Proceedings of the National Academy of Sciences 98, no. 14 (2001): 7736–7741. 10.1073/pnas.131580998 PMC35411 11427712

[49] M. Marin , A. Fernandez , J. Sanchez‐Yagüe , J. Cabezas , and M. Llanillo , “Changes in the Phospholipid and Fatty Acid Composition in Normal Erythrocytes From Sheep of Different Ages. Aminophospholipid Organization in the Membrane Bilayer,” Biochimie 72, no. 10 (1990): 745–750. 2078591 10.1016/0300-9084(90)90159-e

